# Genomic analyses of two *Alteromonas stellipolaris* strains reveal traits with potential biotechnological applications

**DOI:** 10.1038/s41598-018-37720-2

**Published:** 2019-02-04

**Authors:** Marta Torres, Kar-Wai Hong, Teik-Min Chong, José Carlos Reina, Kok-Gan Chan, Yves Dessaux, Inmaculada Llamas

**Affiliations:** 1 Department of Microbiology, Faculty of Pharmacy, University of Granada, Granada, Spain; 2 Institute of Biotechnology, Biomedical Research Center (CIBM), University of Granada, Granada, Spain; 3 Institute for Integrative Biology of the Cell (I2BC), CEA/CNRS/University Paris-Sud, University Paris-Saclay, Gif-sur-Yvette, France; 4 Institute of Biological Sciences, Faculty of Science, University of Malaya, Kuala Lumpur, Malaysia; 5 International Genome Centre, Jiangsu University, Zhenjiang, China

## Abstract

The *Alteromonas stellipolaris* strains PQQ-42 and PQQ-44, previously isolated from a fish hatchery, have been selected on the basis of their strong quorum quenching (QQ) activity, as well as their ability to reduce *Vibrio*-induced mortality on the coral *Oculina patagonica*. In this study, the genome sequences of both strains were determined and analyzed in order to identify the mechanism responsible for QQ activity. Both PQQ-42 and PQQ-44 were found to degrade a wide range of *N*-acylhomoserine lactone (AHL) QS signals, possibly due to the presence of an *aac* gene which encodes an AHL amidohydrolase. In addition, the different colony morphologies exhibited by the strains could be related to the differences observed in genes encoding cell wall biosynthesis and exopolysaccharide (EPS) production. The PQQ-42 strain produces more EPS (0.36 g l^−1^) than the PQQ-44 strain (0.15 g l^−1^), whose chemical compositions also differ. Remarkably, PQQ-44 EPS contains large amounts of fucose, a sugar used in high-value biotechnological applications. Furthermore, the genome of strain PQQ-42 contained a large non-ribosomal peptide synthase (NRPS) cluster with a previously unknown genetic structure. The synthesis of enzymes and other bioactive compounds were also identified, indicating that PQQ-42 and PQQ-44 could have biotechnological applications.

## Introduction

In recent decades, numerous functional genes and enzymes with important industrial applications have been identified in microorganisms using techniques such as high throughput sequencing technologies^1–5^. For instance, whole-genome analyses of many marine bacteria have led to the discovery of a wide range of active metabolites and enzymes of considerable interest for the food, agriculture, aquaculture and pharmaceutical industries^6–10^. Marine microorganisms possess unique properties due to the need to adapt to extreme environmental conditions such as high and low temperatures, alkaline and acidic water, high osmotic stress, high pressure and limited substrate in deep-sea water.

The *Alteromonadaceae* are a family of *Gammaproteobacteria* which currently consists of more than 16 genera (http://www.bacterio.cict.fr)^11^ ubiquitously found in marine environments. Numerous members of the *Alteromonadaceae* family are of biotechnological interest due to their capacity to produce a wide range of metabolites, such as exopolysaccharides (EPSs), as well as antimicrobial and antitumoral agents^12–19^. The genus *Alteromonas* (currently formed by 29 species; http://www.bacterio.cict.fr), one of the most studied and representative members of the *Alteromonadaceae* family, can be found in surface seawater, in the open deep ocean and in coastal seawater. In recent years, the genome sequences of different species of this genus, now available on public databases, have revealed a number of features related to their adaptation to the environment and provide an insight into their potential biotechnological uses^20–23^.

In this study, the genetic and physiological properties of *Alteromonas stellipolaris* strains PQQ-42 and PQQ-44, which were previously isolated from a fish hatchery in Granada in Spain^24^, were analyzed. These isolates showed high quorum quenching (QQ) activity, enabling them to degrade a wide range of *N*-acylhomoserine lactone (AHL) molecules^24^, which are used by many bacteria as signal molecules in cell-to-cell, quorum sensing (QS) communication systems^25^. QS is an efficient mode of intercellular communication in which bacterial gene expression, coupled with bacterial cell concentration, is mediated by the diffusion of specific signal molecules such as the AHLs mentioned above^25^. QS regulates the expression of genes responsible for various phenotypes, including biofilm formation, bioluminescence, conjugal DNA transfer, plasmid copy number control, virulence factors and swarming, all of which have been shown to contribute to bacterial pathogenesis and have a significant impact on human health, aquaculture and agriculture^26,27^. For instance, in many proteobacteria, such as the marine pathogen *Vibrio* spp., AHLs are the principal QS signal molecules^27^ that control the production of virulence factors^28,29^.

The interruption of QS by AHL-degrading QQ enzymes is therefore a promising strategy for controlling bacterial infections in aquaculture^30,31^. In this respect, both strains PQQ-42 and PQQ-44 were previously shown to be capable of degrading AHLs produced by pathogenic *Vibrio* species prone to causing diseases in a wide range of marine animals such as fish, mollusk, crustacean and coral species^24,32–37^. Strains PQQ-42 and PQQ-44 had also been reported to reduce mortality on the coral *Oculina patagonica* infected by *V*. *mediterranei* by quenching both bacterial motility and proteolytic virulence factor production^24^. However, the mechanisms involved in this process had not been identified.

In this study, to gain a better understanding of the lifestyle traits and colony morphologies of strains PQQ-42 and PQQ-44, their genomic sequences were determined and analyzed. Comparative genomics, an essential tool for identifying homologous gene candidates and their functions, were used to detect a possible QQ gene and the genes involved in EPS synthesis in both strains. The production and monosaccharide composition of the EPSs of both strains were characterized, with the presence of rare sugars being detected in PQQ-44. Several bioactive compounds and unusual secondary metabolites were also observed, thus indicating the potential biotechnological applications of these strains.

## Results and Discussion

### Comparison of genomic properties

**Figure 1:**
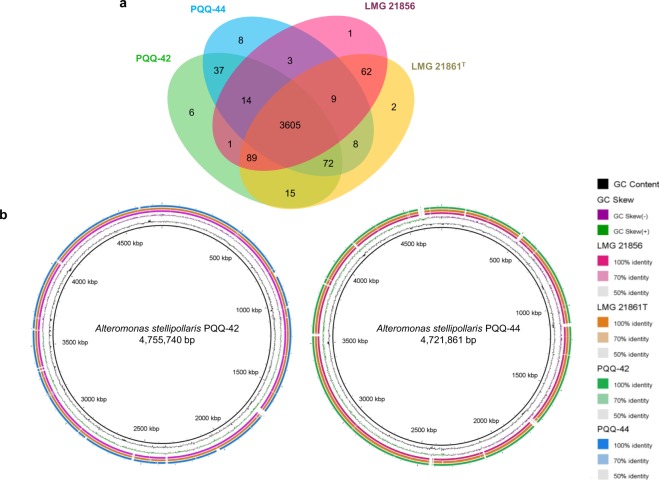
Comparison of the genomes of *Alteromonas stellipolaris* strains PQQ-42, PQQ-44, LMG 21856 and LMG 21861^T^. Venn diagram of orthologous and specific genes in each strain (**a**). BRIG visualization of chromosomal sequences of strains PQQ-42, PQQ-44, LMG 21856 and LMG 21861^**T**^ using PQQ-42 or PQQ-44 as reference sequences (**b**). Gaps in the diagram indicate areas of the genome present in strains PQQ-42 and/or PQQ-44 but absent in the other strains.

**Figure 2:**
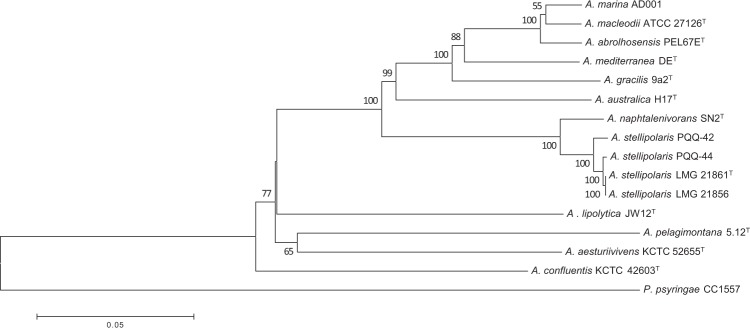
Phylogenetic tree based on concatenated sequences of *Alteromonas* species. The 16S ribosomal RNA, gyrase subunit B gene (*gyrB*) and RNA polymerase subunit beta gene (*rpoB*) were used. The tree was generated using the neighbor-joining method with 1,000 bootstrap replications and *Pseudomonas syringae* CC 1557 as the outgroup.

The genomes of *Alteromonas stellipolaris* strains PQQ-42 and PQQ-44 were 4,755,740 bp and 4,721,860 bp long, respectively, with both having a single chromosome (no plasmid) and a G + C content accounting for 43.6% of the genomes. Of the 4,059 and 4,060 predicted genes in the PQQ-42 and PQQ-44 genomes, 3,978 and 3,979 were protein-coding genes, respectively, with both genomes containing 62 tRNA, 15 rRNA and 3 non-coding RNA. The genome features of all *A*. *stellipolaris* strains whose genomes are available in the NCBI database were compared, namely PQQ-42, PQQ-44, LMG 21856 and LMG 21861^**T**^ (Table 1). 75.50% of the coding DNA sequences (CDSs) of strain PQQ-42 (3,248 CDSs/4,302) and 75.28% of those of strain PQQ-44 (3,250 CDSs/4,317) were found to be in at least one COG group (Table 2). Detailed information on the genomes and composition of both strains is available on the NCBI and MicroScope platforms under accession numbers CP015345.1 and CP015346.1. The genomes of all four *A*. *stellipolaris* strains were compared using the OrthoVenn and BRIG platforms. Based on our OrthoVenn findings, all four strains shared 3,605 genes (Fig. 1a). The BRIG platform revealed that multiple regions in the PQQ-42 and PQQ-44 genomes were absent in the other strains and vice versa (Fig. 1b), suggesting the possible acquisition and/or loss of several additional genes which may have evolutionarily favored their competitiveness in marine environments. To further assess the phylogenetic relatedness of the four strains, the gyrase subunit B gene (*gyrB*), the RNA polymerase subunit beta gene (*rpoB*) and the 16S ribosomal RNA sequences of each *A*. *stellipolaris* strain were concatenated and compared to the sequences of other *Alteromonas* species whose genomes are available in the NCBI database. The phylogenetic tree revealed that the the four *A*. *stellipolaris* strains showed a high level of relatedness among this group of species (Fig. 2). However, based on an analysis of their average nucleotide identity (ANI) values (Table 3), strain PQQ-44 showed a slight evolutionary distance from the other three *A*. *stellipolaris* strains studied.

**Table 1:** Comparison of genomic properties of *Alteromonas stellipolaris* strains PQQ-42, PQQ-44, LMG 21856 and LMG 21861^T^.

Strain	PQQ-42	PQQ-44	LMG 21856	LMG 21861^T^
Isolation source	Fish hatchery	Fish hatchery	Seawater	Seawater
**Accession number**				
Chromosome	CP015345.1 (4,755,740 bp)	CP015346.1 (4,721,861 bp)	CP013120.1 (4,686,200 bp)	CP013926.1 (4,652,019 bp)
Plasmid	n.a.	n.a.	n.a.	CP013927.1 (252,173 bp)
GC content (%)	43.60	43.60	43.60	43.49
Genes	4,059	4,060	4,069	4,193
CDS	3,978	3,979	3,995	4,117
tRNA	62	62	58	57
rRNA	15	15	15	15
ncRNA	3	3	0	3
Number of plasmids	0	0	0	1

The genomic features of each strain were retrieved from the NCBI database.

Legend: n.a., non applicable.

**Table 2:** COG classification of genes in *Alteromonas stellipolaris* strains PQQ-42 and PQQ-44.

Process	Class ID	Description	PQQ-42		PQQ-44		LMG 21856		LMG 21861^T^	
			CDS	%	CDS	%	CDS	%	CDS	%
Information storage and processing	A	RNA processing and modification	1	0.02	1	0.02	1	0.02	1	0.02
Information storage and processing	B	Chromatin structure and dynamics	2	0.05	2	0.05	2	0.05	2	0.05
Metabolism	C	Energy production and conversion	212	4.93	201	4.66	203	4.87	203	4.87
Cellular processes and signaling	D	Cell cycle control, cell division, chromosome partitioning	51	1.19	51	1.18	48	1.15	48	1.15
Metabolism	E	Amino acid transport and metabolism	315	7.32	324	7.51	311	7.45	311	7.45
Metabolism	F	Nucleotide transport and metabolism	73	1.70	75	1.74	73	1.75	73	1.75
Metabolism	G	Carbohydrate transport and metabolism	205	4.77	207	4.80	200	4.79	200	4.79
Metabolism	H	Coenzyme transport and metabolism	130	3.02	132	3.06	128	3.07	128	3.07
Metabolism	I	Lipid transport and metabolism	137	3.18	134	3.10	133	3.19	133	3.19
Information storage and processing	J	Translation, ribosomal structure and biogenesis	194	4.51	193	4.47	189	4.53	189	4.53
Information storage and processing	K	Transcription	270	6.28	267	6.18	259	6.21	259	6.21
Information storage and processing	L	Replication, recombination and repair	180	4.18	186	4.31	163	3.91	163	3.91
Cellular processes and signaling	M	Cell wall/membrane/envelope biogenesis	221	5.14	227	5.26	211	5.06	211	5.06
Cellular processes and signaling	N	Cell motility	132	3.07	128	2.97	126	3.02	126	3.02
Cellular processes and signaling	O	Posttranslational modification, protein turnover, chaperones	145	3.37	151	3.50	147	3.52	147	3.52
Metabolism	P	Inorganic ion transport and metabolism	251	5.83	251	5.81	250	5.99	250	5.99
Metabolism	Q	Secondary metabolite biosynthesis, transport and catabolism	90	2.09	84	1.95	89	2.13	89	2.13
Poorly characterized	R	General function prediction only	539	12.53	542	12.56	531	12.73	531	12.73
Poorly characterized	S	Function unknown	281	6.53	284	6.58	276	6.62	276	6.62
Cellular processes and signaling	T	Signal transduction mechanisms	315	7.32	312	7.23	307	7.36	307	7.36
Cellular processes and signaling	U	Intracellular trafficking, secretion, and vesicular transport	113	2.63	112	2.59	111	2.66	111	2.66
Cellular processes and signaling	V	Defense mechanisms	58	1.35	56	1.30	55	1.32	55	1.32
Cellular processes and signaling	W	Extracellular structures	4	0.09	3	0.07	4	0.10	4	0.10
Cellular processes and signaling	Z	Cytoskeleton	1	0.02	2	0.05	1	0.02	1	0.02

Clusters of orthologous gene groups were retrieved from the MicroScope database.

**Table 3:** Average nucleotide identity (ANI) values for species of the genus *Alteromonas*.

	KCTC 52655^T^	PQQ-44	PQQ-42	LMG 21861^T^	LMG 21856	AD001	9a2^T^	KCTC 42603^T^	JW12^T^	SN2^T^	ATCC 27126^T^	5.12^T^	H17^T^	DE^T^	PEL67E^T^
*A*. *aesturiivivens* KCTC 52655^T^	—														
*A*. *stellipolaris* PQQ-44	69.74	—													
*A*. *stellipolaris* PQQ-42	69.60	98.94	—												
*A*. *stellipolaris* LMG 21861^T^	69.67	98.85	99.24	—											
*A*. *stellipolaris* LMG 21856	69.63	98.89	99.30	99.91	—										
*A*. *marina* AD001	69.72	73.41	73.42	73.48	73.47	—									
*A*. *gracilis* 9a2^T^	70.09	73.42	73.41	73.36	73.42	80.97	—								
*A*. *confluentis* KCTC 42603^T^	70.49	69.77	69.75	69.73	69.76	70.13	69.86	—							
*A*. *lipolytica* JW12^T^	69.95	69.22	69.23	69.21	69.26	69.57	69.40	69.90	—						
*A*. *naphtalenivorans* SN2^T^	69.59	89.63	89.63	89.47	89.63	73.64	73.25	69.68	69.13	—					
*A*. *macleodii* ATCC 27126^T^	69.90	73.40	73.38	73.46	73.45	90.10	80.71	69.90	69.43	73.47	—	—			
*A*. *pelagimontana* 5.12^T^	70.49	69.69	69.88	69.72	69.79	69.97	69.81	70.05	69.22	70.17	69.72	69.72	—		
*A*. *australica* H17^T^	69.75	73.45	73.45	73.52	73.56	73.84	73.61	69.65	69.14	73.50	73.69	73.69	69.78	—	
*A*. *mediterranea* DE^T^	69.97	73.54	73.53	73.48	73.57	80.74	79.90	70.28	69.70	73.74	80.69	80.69	70.39	74.43	—
*A*. *abrolhosensis* PEL67E^T^	69.74	73.50	73.52	73.43	73.48	86.50	80.50	69.94	69.51	73.51	88.40	88.40	69.72	73.87	80.60

### Cell/colony morphology and cell wall properties

**Figure 3:**
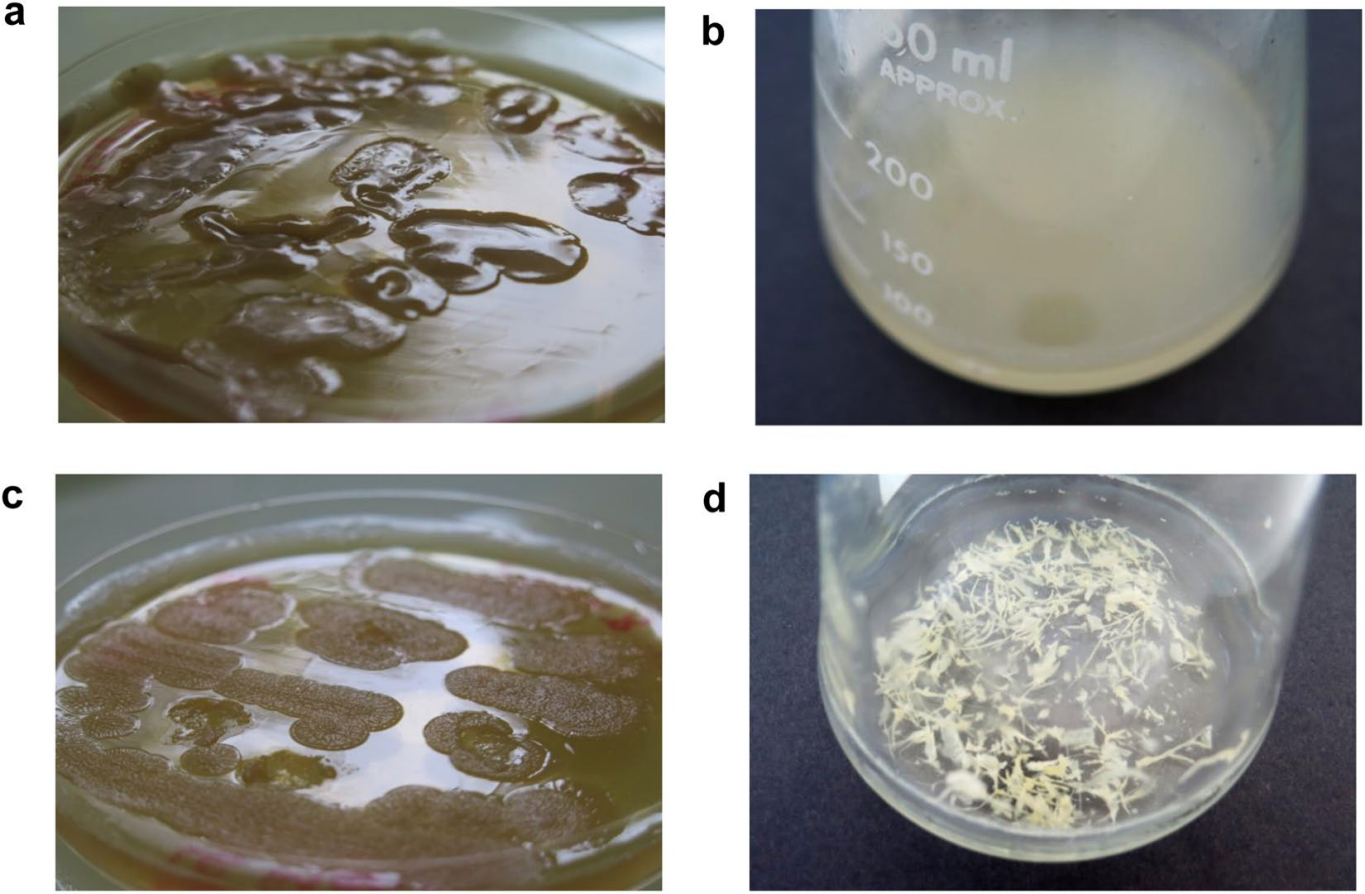
Growth differences in *Alteromonas stellipolaris* PQQ-42 and PQQ-44 in solid and liquid medium. Colony morphology and growth appearance of strains PQQ-42 (**a**,**b**) and PQQ-44 (**c**,**d**) after 24 hours of incubation in MA and MB.

Interestingly, strains PQQ-42 and PQQ-44 exhibited very distinct phenotypes. On solid media, strain PQQ-42 produced mucoid, smooth colonies, while strain PQQ-44 formed dry, rough colonies. In liquid media, PQQ-42 grew homogeneous, while PQQ-44 produced aggregates, thus confirming their genotypic and physiological differences (Fig. 3). As with other bacteria, this dissimilarity could be explained by differences in cell wall properties and composition^38–40^. Thus, using MAUVE software, the following genes involved in cell wall synthesis and shape determination were detected only in strain PQQ-42: the *wecB* gene encoding UDP-N-acetylglucosamine 2-epimerase, *wecC* encoding UDP-N-acetyl-D-mannosaminuronic acid dehydrogenase and gene *mreB* encoding a rod shape-determining protein (MreB). On the other hand, genes encoding mannan endo-1,4-beta-mannosidase, beta-mannanase, mannose-1-phosphate guanylyl-transferase, mannose-6-phosphate isomerase, GDP-mannose mannosyl hydrolase and GDP-fucose synthetase were identified only in strain PQQ-44. Following a KEGG pathway comparison of the two strains, these genes were found to be involved in amino/nucleotide sugar and fructose/mannose metabolisms. These features may explain some of the differences in the growth phenotypes of strains PQQ-42 and PQQ-44.

**Figure 4:**
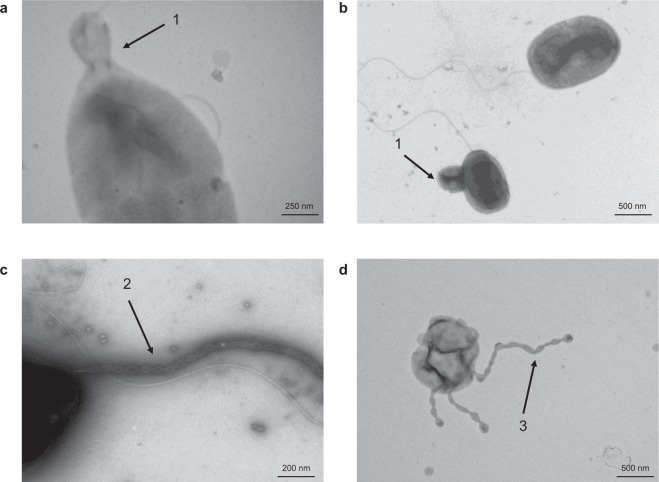
Budding division of strains PQQ-42 and PQQ-44. Observation by transmission electron microscopy of emerging buds (1), bud chains (2) and prosthecae (3) in strains PQQ-42 (**a**,**d**) and PQQ-44 (**b**,**c**), as indicated by arrows.

Despite colony morphology and cell wall differences, both PQQ-42 and PQQ-44 (which exhibit buds, cell chains and prosthecae) showed similar budding patterns when observed by transmission electron microscopy (Fig. 4). This cell division mechanism has been previously described for *Alteromonas stellipolaris* type strain LMG 21861^T^, a budding prosthecate bacterium which is motile by a single, polar flagellum^41^. In binary fission, bacteria grow symmetrically and divide and produce two identical sibling cells, while, in budding division, the new offspring emerges *de novo* from a morphologically invariant mother cell. In some cases, although offspring can differ from their mother cell, no differences in cell wall composition have been reported so far with respect to budding division.

### Exopolysaccharide production and chemical composition

**Figure 5:**
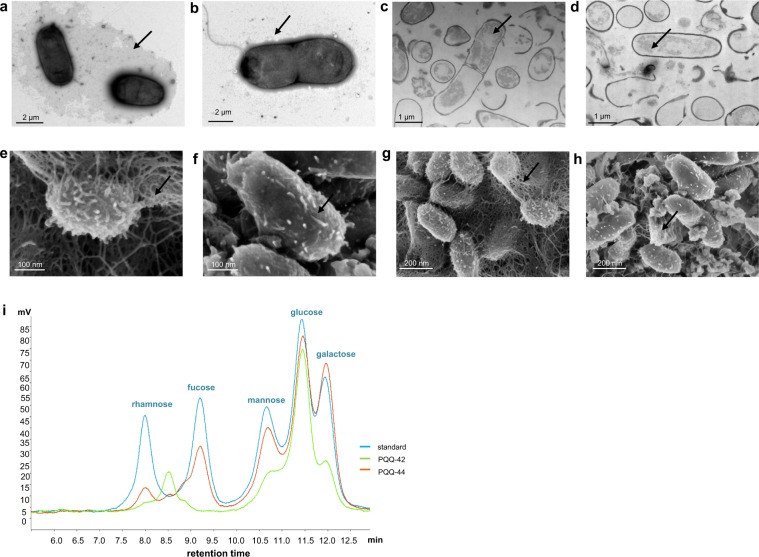
Exopolysaccharide production in strains PQQ-42 and PQQ-44. Uranyl acetate staining of strains PQQ-42 (**a**) and PQQ-44 (**b**) as observed by transmission electron microscopy. Ruthenium red staining of strains PQQ-42 (**c**) and PQQ-44 (**d**) as observed by transmission electron microscopy. Strains PQQ-42 (**e**,**g**) and PQQ-44 (**f**,**h**) as observed by scanning electron microscopy after critical point drying. Arrows indicate exopolysaccharide accumulation in each strain. Monosaccharide composition of exopolysaccharides produced by strains PQQ-42 and PQQ-44 determined by ion chromatography (**i**).

As for other bacteria, differences in the colony morphologies of strains PQQ-42 and PQQ-44 can also be explained by dissimilarities in EPS production and composition^38–40^. Using scanning and transmission electron microscopy, both strains were found to produce EPSs located inside the cells and in the surrounding medium, although EPS production appeared to be higher in strain PQQ-42 (Fig. 5). Likewise, several genes associated with EPS synthesis and transport were found in the genomes of both *A*. *stellipolaris* strains PQQ-42 and PQQ-44. Moreover, the EPSs, which were synthesized mainly during the early stationary growth phase under our experimental conditions, were extracted and gravimetrically quantified after the bacteria were grown under optimal conditions for five days. The EPS yield was higher in strain PQQ-42 (0.36 g l^−1^) than in strain PQQ-44 (0.15 g l^−1^), which confirmed the results observed by scanning electron microscopy. These EPS production values are similar to those obtained with other marine bacteria^42,43^.

To obtain additional information on the differences between their EPSs, the monosaccharide content of strains PQQ-42 and PQQ-44 was determined using ion chromatography (Fig. 5i). The EPS of strain PQQ-42 was found to be composed of 122.93 mg l^−1^ glucose, 45.60 mg l^−1^ galactose, 43.55 mg l^−1^ mannose, 7.16 mg l^−1^ rhamnose and another unidentified sugar, which was neither arabinose nor trehalose, sugars which have a similar retention time. Strain PQQ-44 produced an EPS with a very different monosaccharide composition: 144.24 mg l^−1^ galactose, 130.69 mg l^−1^ glucose, 82.36 mg l^−1^ mannose and 24.95 mg l^−1^ rhamnose. Remarkably, its EPS also contained 74.03 mg l^−1^ fucose (Fig. 5i), a sugar not commonly found in bacterial EPSs. Moreover, some genes encoding functions associated with fucose synthesis, such as GDP-mannose mannosyl hydrolase and GDP-L-fucose synthetase^44^, were present only in the PQQ-44 genome (data not shown). Rare sugars, such as fucose, may provide EPS with additional biological properties as compared to those composed of more common sugar monomers^45^. Fucose and fucose-containing oligosaccharides play a high-value role in sectors related to cosmetics, food products, pharmaceuticals and biomedicine. Their potential applications include: prevention of tumor cell lung colonization, regulation of white blood cell formation, rheumatoid arthritis treatment and antigen synthesis for antibody production^46,47^. Fucose, which is often in short supply, is usually obtained through chemical synthesis or algae extraction, which are arduous and expensive processes. As reported for EPSs excreted by *Salipiger mucosus*, *Klebsiella pneumoniae* and *Clavibacter michiganensis*^47,48^, EPS from strain PQQ-44 may therefore prove to be a cheaper and more straightforward source of this rare sugar. In addition, both EPS aqueous solutions, with their low viscosity (2,300 mPa s^−1^ for PQQ-42 and 1,800 mPa s^−1^ for PQQ-44), pseudoplastic behavior and ability to jellify in acid medium, offer a wide range of potential biotechnological applications.

### Quorum quenching properties

In a previous study^24^, strains PQQ-42 and PQQ-44 were shown to actively degrade a range of synthetic AHLs (such as C8-HSL, C10-HSL, 3-OH-C10-HSL, C12-HSL, 3-O-C12-HSL and 3-OH-C12-HSL; see abbreviations in experimental procedures) used as QS signals by numerous proteobacteria^24^ like the pathogenic species *Vibrio anguillarum* ATCC 19264^T^, *V*. *nigripulchritudo* CIP 103195^T^, *V*. *metschnikovii* NCTC 8483^T^, *V*. *mediterranei* VibC-Oc-097, *V*. *coralliilyticus* VibC-Oc-193 and *V*. *owensii* VibC-Oc-106^24,32,37^.

**Figure 6:**
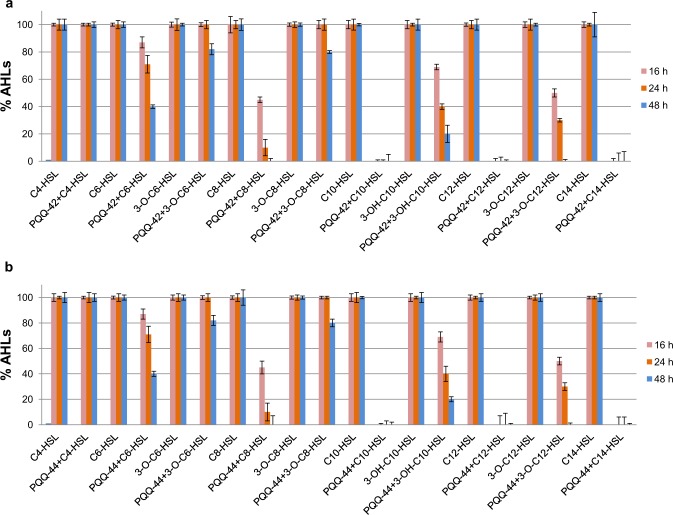
Time-dependent AHL-degradation of synthetic AHLs by strains PQQ-42 and PQQ-44. The graph shows the percentage of signal molecules remaining after 16, 24 and 48 hours of incubation with strain PQQ-42 (**a**) or PQQ-44 (**b**). Error bars represent standard deviations.

In this study, to further characterize the QQ properties of both strains, their time-dependent AHL degradation capacity was assessed after 16, 24 and 48 hours of incubation using various synthetic AHL molecules. To conduct these experiments, the following AHL molecules, including those already tested in a previous study^24^ as well as new types of AHLs, were used: C4-HSL, C6-HSL, 3-O-C6-HSL, C8-HSL, 3-O-C8-HSL, C10-HSL, 3-OH-C10-HSL, C12-HSL, 3-O-C12-HSL and C14-HSL. The novel results obtained (Fig. 6) show that both strains had similar time-dependent degradation capacities and that their QQ activity was stronger and more rapid against the long-chain, unsubstituted AHLs C10-HSL, C12-HSL and C14-HSL between 0 and 16 hours of incubation, as shown by the total degradation of the molecules tested. The medium-chain, chemically-substituted AHLs C8-HSL and 3-O-C12-HSL were fully degraded after 48 hours of incubation. However, the shorter-chain AHLs were degraded less efficiently. After a 48-hour period of incubation, 60% of C6-HSL was degraded, while only 20% of 3-O-C6-HSL was degraded under these conditions. Our study also confirms a previous finding that the short-chain C4-HSL cannot be degraded^24^ even after 48 hours of incubation by any of the two QQ strains.

**Figure 7:**
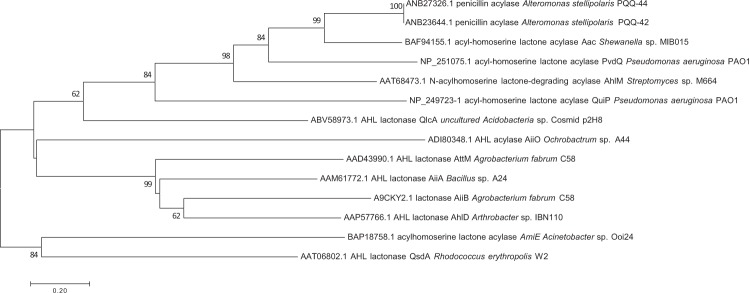
Phylogenetic analysis of the penicillin acylase of PQQ-42 and PQQ-44. Tree generated by Neighbor-Joining method with 1,000 bootstrap replications and based on known acylase and lactonase aminoacid sequences.

According to previous experimental results^24^, the AHL degradation products were not converted back to intact AHLs during an acidification assay^49^. Therefore, the QQ activity of the two selected strains may not be caused by an acyl homoserine-yielding lactonase but rather by an AHL acylase (or amidohydrolase), which releases a homoserine lactone and a fatty acid. In this regard, we found that the penicillin acylase genes ANB23644.1 and ANB27326.1 encode an 859-amino acid protein in the PQQ-42 and PQQ-44 genomes, respectively. As with most AHL acylases described to date^50^, the deduced proteins belong to the Ntn-hydrolase superfamily. They show 62% similarity and 49% identity to the AHL-acylase (amidohydrolase) Aac from *Shewanella* sp. MIB015 (BAF94155.1), 32% similarity and 48% identity to the AHL-acylase PvdQ from *Pseudomonas aeruginosa* PAO1 (NP_251075.1), as well as 31% similarity and 46% identity to the AHL-acylase AhlM of *Streptomyces* sp. M664 (AAT68473.1), all of which have a proven capacity to degrade different types of AHLs^51–53^. The deduced proteins also exhibit similarity to the proteins of other marine genera of the *Alteromonadaceae* family; these include acylases in *Alteromonas nadita*, *A*. *naphtalenivorans*, *A*. *australica*, *A*. *marina*, *A*. *macleodii*, *A*. *mediterranea*, *Salinimonas chungwensi*, *Glaciecola pallidula* and *Thalassotalea* sp. strain PP2-459, a marine QQ bacterium previously studied in our laboratory^54^, thus indicating that acylases are frequently present in marine bacteria. These results were confirmed by phylogenetic analysis (Fig. 7) where the QQ enzymes of *A*. *stellipolaris* PQQ-42 and PQQ-44 cluster in the acylase rather than the lactonase clade.

This is not the first time that strains of *A*. *stellipolaris* have been screened for AHL-degrading activity. *A*. *stellipolaris* strain PP2-67, which was isolated from a mollusk hatchery^54^, has recently been shown to degrade synthetic AHLs, although its activity is less significant than that of strains PQQ-42 and PQQ-44 (data not shown).

The actual physiological significance of AHL-degrading enzymes remains largely unclear^50,55^. As observed with other bacteria, some authors have suggested that AHL degradation could be associated with a self-regulation of intercellular systems^53,56,57^. Using different AHL biosensor strains, we found that neither PQQ-42 nor PQQ-44 appear to produce any AHLs, and could not identify any gene coding for a functional QS signal synthase in their genomes. This demonstrates that the AHL-degrading capacity of strains PQQ-42 and PQQ-44 is not related to self-regulating intercellular systems.

### Antimicrobial metabolite biosynthesis

**Figure 8:**
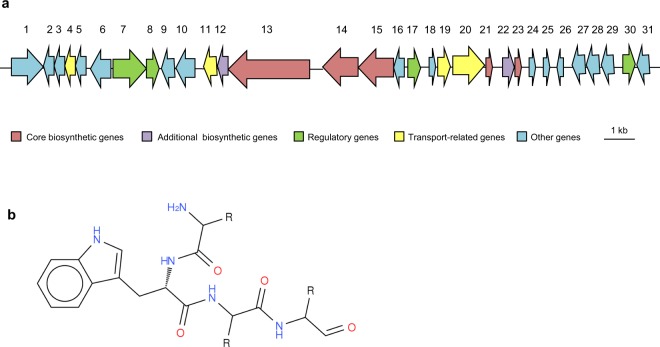
PQQ-42 non-ribosomal peptide synthase gene cluster overview. Biosynthetic, transport and regulation genes (**a**) and predicted structure of the non-ribosomal peptide encoded in the PQQ-42 genome (**b**). Gene number and associated predicted function are as follows: 1, α-galactosidase, 2, tetratricopeptide; 3, acetylhydrolase/lipoprotein signal peptide; 4, major facilitator superfamily MFS1; 5, dihydrodipicolinate synthase; 6, hypothetical protein; 7, ATP-binding region, ATPase-like protein/hybrid sensor histidine kinase/response regulator; 8, response regulator receiver protein; 9, hypothetical protein; 10, hypothetical protein; 11, ABC-type siderophore export system, fused ATPase and permease/cyclic peptide transporter; 12, SyrP-like protein; 13, non-ribosomal peptide synthase; 14, non-ribosomal peptide synthetase; 15, long-chain-fatty-acid-CoA ligase/non-ribosomal peptide synthase; 16, hypothetical protein; 17, transcriptional regulator, CadC; 18, hypothetical protein; 19, membrane fusion protein of RND family multidrug efflux pump; 20, acriflavin resistance protein; 21, thioesterase; 22, vulnibactin synthase, phosphopantetheinyl transferase component; 23, hypothetical protein; 24, hypothetical protein; 25, ISXo8 transposase; 26, single-stranded DNA-binding protein; 27, putative sodium-dependent galactose transporter; 28, galactokinase; 29, galactose-1-phosphate uridylyltransferase; 30, galactose mutarotase; 31, galactose operon repressor, GalR-LacI family of transcriptional regulators.

Genome mining has been used to identify a wide range of novel secondary metabolites exhibiting pharmacologic activity in marine microorganisms, with many more to be discovered in the future which can be used either directly as drugs or as templates for chemical drug synthesis^8,58–60^. We mined the PQQ-42 and PQQ-44 genomes using antiSMASH, a bioinformatic tool used to identify clusters involved in secondary metabolite synthesis. In both strains siderophore and bacteriocin synthesis gene clusters were identified (data not shown). It was also found that strain PQQ-42 harbors a large non-ribosomal peptide synthase (NRPS) cluster (54,167 bp). This cluster, whose unusual genetic structure is reported for the first time, is composed of core NRPS genes surrounded by other coding determinants. These include the encoding of lactose and galactose uptake and degradation, multidrug resistance efflux pumps and flagellar motility systems (Fig. 8a). The antiSMASH software pipeline was used as well to predict the putative chemical structure of the molecule produced by this gene cluster (Fig. 8b).

In order to identify the molecule produced by strain PQQ-42, the largest biosynthetic gene in the NRPS cluster, ORF 13, was mutated. Using HPLC/MS, the products produced by the mutant were compared to those produced by the wild-type strain, and no significant differences were observed.

### Hydrolytic enzymes and vitamin synthesis

Given that microbial communities in marine ecosystems have been reported to produce enzymes with biotechnological applications^61,62^, several enzymatic determinants which could have a wide-range of practical applications were identified in PQQ-42 and PQQ-44 genome sequencing. These include genes encoding alginate lyases, used to produce biofuel^63^ and to remove persistent alginate biofilms in clinical samples and medical devices^64,65^; α-amylases, used in a wide variety of industrial processes^66^; and chitinases for the pharmaceutical industry, chitinous waste treatment and mosquito control^67,68^. However, these three enzymatic activities of PQQ-42 and PQQ-44, which were tested and detected *in vitro* (data not shown), need to be studied in greater depth. Other genes encoding enzymes, including lipases agarases, pectate lyases and trehalases, with several biotechnological applications, have also been found in the genomes of both *Alteromonas* strains.

In addition, strains PQQ-42 and PQQ-44 appear to be able to synthesize several vitamins, as we found that determinants for most enzymes needed for the synthesis of biotin, riboflavin and thiamine are present in their genomes.

### General stress response

To survive in marine environments, *Alteromonas stellipolaris* strains PQQ-42 and PQQ-44 need to withstand different stress conditions, a feature that may improve their potential use in aquaculture. Moreover, in recent years, the possible application of stress proteins in anticancer therapeutics and the food industry has been investigated^69,70^. Genes related to general stress were identified in the genomes of the two bacteria selected, including those encoding the starvation stringent protein (*Ssp*) of *E*. *coli*, the RNA polymerase sigma factor RpoH and three phage shock proteins PspA, PspB and PspC. Genes coding for choline dehydrogenase (BetA) and choline sulfatase (BetC), two enzymes involved in the synthesis of glycine betaine, an industrially important osmoprotectant, were also found in the genomes studied. Determinants for other enzymes associated with oxidative stress, including catalase, superoxide dismutase and alkyl hydroperoxide reductase, were identified as well. Cold shock protein genes *cspA*, *cspD* and *cspG*, as well as genes encoding DnaK, DnaJ and GrpE chaperones which may protect PQQ-42 and PQQ-44 against heat shock and oxidative stress, were also detected. These stress and heat shock proteins are of considerable importance in the pharmaceutical industry, where they are used in cancer vaccines and inmunotherapy^71^.

### Motility

**Figure 9:**
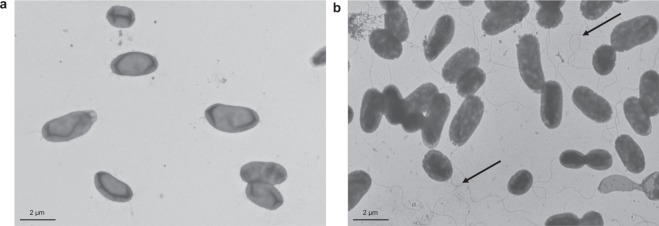
Flagella observed by transmission electron microscopy. Absence of flagellum in strain PQQ-42 (**a**) and polar flagellum in strain PQQ-44 (**b**), as indicated by arrow.

The motility of strains PQQ-42 and PQQ-44 used as biological inputs in aquaculture, which facilitate the dispersal of bacteria in aquaculture and septic tank facilities, could be an additional benefit. However, if the *A*. *stellipolaris* strains needed to be immobilized in plastic floatation tank devices, motility would not be a critical requirement. In this respect, up to 44 flagella-related genes, including *flaA*, *fleN*, *fleQ*, *fleS*, *fliE*, *fliF*, *fliG*, *fliH*, *fliI*, *fliK*, *fliL*, *fliM*, *fliN*, *fliQ*, *fliR*, *flhA*, *flhB* and *flhF*, have been identified in the genome of both PQQ-42 and PQQ-44. However, when tested *in vitro*, only strain PQQ-44 exhibited swimming motility in plate assays (data not shown) and a polar flagellum under a transmission electron microscope (Fig. 9). Both strains actually contained the same number of components required for flagellum synthesis and assembly. However, they differed in relation to the flagellar hook-length control protein FliK, with a variation being observed in the aminoacid number of two out of the three FliK proteins encoded in the genomes of both strains. One of the *fliK* genes in strain PQQ-42 encoded an 837-aa protein, while its ortholog in strain PQQ-44 encoded an 840-aa protein. By contrast, for another FliK protein, the expected length of the deduced protein in strain PQQ-42 was 766 aa, but 762 aa for the cognate protein in strain PQQ-44. Although the role of these proteins remains unclear^72^, FliK appears to determine the minimal length of the hook and also acts as an essential checkpoint controller. FliK detects when flagellar hook assembly is complete, terminates this formative stage and then triggers the filament export stage in the biosynthetic pathway^73^. The FliK proteins of PQQ-42 may lead to the inactivation of flagellar biosynthesis or assembly, which accounts for this strain’s nonmotile phenotype. One possible explanation for the difference in motility between the two strains may be related to the mechanism involved in their cell division through budding, as, in some cases, offspring can differ from their mother cell through the gain or loss of flagellar systems^74^.

## Conclusions

Current studies of *Alteromonas* genomes focus on identifying the ecological niche, adaptation and geographic distribution of species. In this study, we identified the different traits which typify strains PQQ-42 and PQQ-44 and endow them with considerable biotechnological potential. These features include the production of numerous enzymes, such as the acylase, possibly responsible for the high quorum quenching activity; the rare fucose-rich exopolysaccharides produced by strain PQQ-44 and the potential to synthesize novel secondary metabolites.

## Experimental Procedures

### Bacterial growth, enzyme assays and DNA extraction

*Alteromonas stellipolaris* strains PQQ-42 and PQQ-44, which had been previously isolated from the seawater of a fish rearing hatchery in Granada (Spain, 36°44′44.2′′N, 3°36′04.8′′W)^24^, were routinely grown on marine broth (MB, Difco), marine agar (MA, Difco) and sterile filtered seawater with 0.1% (w/v) yeast extract (SFSWYE) at 25 °C.

The biosensors *Chromobacterium violaceum* CV026^75^ and *Agrobacterium tumefaciens* NTL4 (pZLR4)^76^ were used to detect substituted and non-substituted, short-chain (C4 and C6) and medium- to long-chain (C8 to C14) AHL molecules, respectively. Both biosensors were grown on Luria-Bertani (LB) medium at 28 °C, and, when required, the antibiotics kanamycin and gentamicin were used at a final concentration of 50 μg ml^−1^.

To test for chitinase activity, strains PQQ-42 and PQQ-44 were grown on MA supplemented with 1% (w/v) colloidal chitin^77^. α-amylase activity was assessed on MA supplemented with 1% (w/v) starch^78^. Alginate hydrolysis was determined on MA supplemented with 0.75% (w/v) sodium alginate^79^. In all these media, the results were obtained by measuring haloes around the spotted area after a 7-day incubation period at 25 °C.

Genomic DNA was extracted from 5 ml overnight cultures using the MasterPure Complete DNA Purification kit (Epicentre) according to the manufacturer’s instructions. Genomic DNA was visualized on 0.8% (w/v) agarose gels stained with RedSafe (iNtRON Biotechnology) and quantified using a Nanodrop microvolume spectrophotometer (Thermo Fisher).

### Genome sequencing, annotation, analysis and comparison

The genome sequences of *A*. *stellipolaris* PQQ-42 and PQQ-44 were determined with the aid of an RSII sequencer using single-molecule real-time (SMRT) sequencing technology (Pacific Biosciences). The genomic DNA libraries were constructed using the Template Preparation and P6 DNA Polymerase Binding kits (Pacific Biosciences). After sequencing, the reads were assembled using the hierarchical genome-assembly process (HGAP) software v.3.0^80^. Annotation was performed using the NCBI prokaryotic genome annotation pipeline (PGAP)^81^. The genomes of both strains were deposited in GenBank under accession numbers CP015345.1 and CP015346.1. The genome sequences of PQQ-42, PQQ-44, LMG 21856 and LMG 21861^**T**^ were compared using the OrthoVenn, MAUVE and BLAST Ring Image Generator (BRIG) platforms^82–84^. Sequence alignment and phylogenetic analyses were carried out with the aid of MEGA7 software^85^. The microbial genome annotation and analysis platform MicroScope enabled us to identify clusters of orthologous gene groups^86^. The average nucleotide identity (ANI) value was obtained according to guidelines described by Konstantinidis and Tiedje^87^. The metabolic pathways were compared using the KEGG database^88^.

### AHL degradation bioassay

The following synthetic AHLs (Sigma-Aldrich) were used at a final concentration of 25 μM to evaluate the AHL degradation activity of PQQ-42 and PQQ-44: C4-HSL (*N*-butyryl-DL-homoserine lactone), C6-HSL (*N*-hexanoyl-DL-homoserine lactone), 3-O-C6-HSL (*N*-3-oxo-hexanoyl-DL-homoserine lactone), C8-HSL (*N*-octanoyl-DL-homoserine lactone), 3-O-C8-HSL (*N*-3-oxo-octanoyl-DL-homoserine lactone), C10-HSL (*N*-decanoyl-DL-homoserine lactone), 3-OH-C10-HSL (*N*-3-hydroxydecanoyl-DL-homoserine lactone), C12-HSL (*N*-dodecanoyl-DL-homoserine lactone), 3-O-C12-HSL (*N*-3-oxo-dodecanoyl-DL-homoserine lactone) and C14-HSL (*N*-tetradecanoyl-DL-homoserine lactone). Briefly, cultures of the two *A*. *stellipolaris* strains (OD_600_ 1.5) were mixed with AHLs at the above mentioned final concentration. The mixtures (500 µl of culture supplemented with 0.5 µl of each synthetic AHL) were incubated at 25 °C for 16, 24 and 48 hours, and the remaining AHLs were detected using a well diffusion agar-plate assay technique described elsewhere^24^ with the aid of the biosensors *C*. *violaceum* CV026 and *A*. *tumefaciens* NTL4 (pZLR4). The diameters of the colored haloes were measured and compared to controls to determine the percentage of signal molecules remaining in each case. These assays were carried out in triplicate.

### Identification of secondary metabolite biosynthetic genes

The potential capacity of the two *A*. *stellipolaris* strains to produce antagonistic compounds was analyzed *in silico* using the antiSMASH database of microbial secondary metabolite biosynthetic gene clusters^89^. The mutation of the NRPS gene cluster was created as follows: a 355-bp ORF 13 internal fragment in the NRPS cluster was amplified from strain PQQ-42 using the m13E forward primer 5′-CGCGAATTCCCAAGGCAGATGGCAGCACT-3′ and m13X reverse primer 5′-GCGTCTAGAGCGTTAATCGAGTTACTAA GAG-3′ which include *Eco*RI and *Xba*I restriction sites (underlined) to facilitate cloning in the suicide vector pVIK112^90^. The construction was transformed into S17 λ*pir* and transferred to a rifampicin-resistant PQQ-42 derivative by biparental mating, involving gene replacement by single recombination^91^. To determine whether the selected clones contained the plasmid, PCR was conducted using the NRPS13 forward primer 5′-ATGAAAATGCAGCACATTATTG-3′ and *lacZ* reverse primer 5′-GCTTCATCAGGATATCC-3′, which gave a 1,500 bp fragment. The wild type and mutant strains were analyzed by HPLC/MS in order to identify the molecule produced by the NRPS gene cluster. Briefly, filtered supernatant of 5 ml cultures of each strain in MB and SFSWYE media at an early exponential phase (OD_600_ 0.8) and middle stationary phase (OD_600_ 2.8) were used. HPLC/MS analyses were carried out under the conditions described by Juguet *et al*.^92^.

### Exopolysaccharide production and monosaccharide content analysis

The exopolysaccharides produced by strains PQQ-42 and PQQ-44 were isolated using the method described by Quesada *et al*.^93^. Briefly, the strains were grown in MB supplemented with 1% (w/v) glucose at 25 °C and shaken in a rotary shaker at 100 rpm to a maximum OD_600_ of 2.2. The cultures were then centrifuged at 4,000 × *g* for 20 minutes, and the supernatant was precipitated with cold ethanol for 16 hours at 4 °C, centrifuged under conditions similar to those described above, dialyzed against distilled water and finally lyophilized^94^. Dilutions of 1% (w/v) EPS in distilled water were prepared and measured in a rheometer at room temperature to analyze the rheological properties of EPSs. Dilutions of 0.5% (w/v) EPS in distilled water were acidified to pH 3 and visually checked for gelification in order to test its capacity to jellify in acid medium. Monosaccharides were quantitatively determined by ion chromatography after EPS was treated with 0.9 M methanolic HCl for 16 hours at 80 °C^95^.

Exopolysaccharides were observed with the aid of a scanning electron microscope (GeminiSEM, Zeiss, Germany) using the critical point drying method, as well as under a transmission electron microscope (LEO906E, Zeiss, Germany) using overnight cultures of strains PQQ-42 and PQQ-44. For observation by transmission electron microscope, cells were negatively stained with 1% (w/v) uranyl acetate (pH 7.4) or 0.1% (w/v) ruthenium red on a Formvar carbon-coated grid.

### Flagella observation and motility assay

Flagella in strains PQQ-42 and PQQ-44 were observed using a transmission electron microscope (LEO906E, Zeiss, Germany) after negatively staining overnight bacterial cultures with 1% (w/v) uranyl acetate (pH 7.4). Swimming motility was evaluated by inoculating both strains on MA plates containing 0.3% (w/v) agar^96^. The plates were analyzed after incubation at 25 °C for approximately 24 hours. Growth due to the migration of cells from the inoculation site was measured. The experiments were performed in triplicate.
